# High Prevalence of Abdominal, Intra-Abdominal and Subcutaneous Adiposity and Clustering of Risk Factors among Urban Asian Indians in North India

**DOI:** 10.1371/journal.pone.0024362

**Published:** 2011-09-20

**Authors:** Swati Bhardwaj, Anoop Misra, Ranjita Misra, Kashish Goel, Surya Prakash Bhatt, Kavita Rastogi, Naval K. Vikram, Seema Gulati

**Affiliations:** 1 National Diabetes, Obesity and Cholesterol Diseases Foundation (N-DOC), SDA, New Delhi, India; 2 Diabetes Foundation (India), SDA, New Delhi, India; 3 Fortis CDOC Center for Excellence for Diabetes, Metabolic Diseases and Endocrinology, Fortis Flt. Lt. Rajan Dhall Hospital, New Delhi, India; 4 Center for Internal Medicine, Fortis Flt. Lt. Rajan Dhall Hospital, New Delhi, India; 5 Department of Health and Kinesiology, Texas A&M University, College Station, Texas, United States of America; 6 Department of Internal Medicine, Wayne State University, Detroit, Michigan, United States of America; 7 Department of Biochemistry and Medicine, All India Institute of Medical Sciences, New Delhi, India; 8 Department of Internal Medicine, All India Institute of Medical Sciences, New Delhi, India; Universita Magna-Graecia di Catanzaro, Italy

## Abstract

**Objective:**

To assess the prevalence of abdominal obesity including intra-abdominal and subcutaneous adiposity along with other cardiometabolic risk factors in urban Asian Indians living in New Delhi.

**Methods:**

We conducted a cross-sectional epidemiological descriptive study with 459 subjects (217 males and 242 females), representing all socio-economic strata in New Delhi. The anthropometric profile [body mass index (BMI), waist circumference (WC) and skinfold thickness], fasting blood glucose (FBG) and lipid profile were recorded. Percent body fat (%BF), total abdominal fat (TAF), intra-abdominal adipose tissue (IAAT) and subcutaneous abdominal adipose tissue (SCAT) were quantified using predictive equations for Asian Indians.

**Results:**

The overall prevalence of obesity was high [by BMI (>25 kg/m^2^), 50.1%]. The prevalence of abdominal obesity (as assessed by WC) was 68.9%, while that assessed by TAF was 70.8%. Increased IAAT was significantly higher in females (80.6%) as compared to males (56.7%) (p = 0.00) with overall prevalence being 69.3%. The overall prevalence of high SCAT was 67.8%, more in males (69.1%) vs. females (66.5%, p = 0.5). The prevalence of type 2 diabetes, the metabolic syndrome and hypertension was 8.5%, 45.3% and 29.2%, respectively. Hypertriglyceridemia, hypercholesterolemia and low levels of HDL-c were prevalent in 42.7%, 26.6% and 37% of the subjects, respectively. The prevalence of hypertriglyceridemia was significantly higher in males (p = 0.007); however, low levels of HDL-c were more prevalent in females as compared to males (p = 0.00).

**Conclusion:**

High prevalence of generalized obesity, abdominal obesity (by measurement of WC, TAF, IAAT and SCAT) and dysmetabolic state in urban Asian Indians in north India need immediate public health intervention.

## Introduction

Obesity is an increasingly important health problem worldwide including the developing countries like India [1]. Obesity, abdominal obesity, and co-morbidities are increasingly prevalent among urban Indians [1]. Regional fat distribution, particularly abdominal obesity, is considered important for development of insulin resistance, the metabolic syndrome and coronary heart disease [2].

More than 80% of total body fat is distributed in the subcutaneous adipose tissue (SCAT) and 10–20% within visceral/intra-abdominal adipose tissue (IAAT) in adults [3]. The two major abdominal adipose tissue depots: IAAT and SCAT have been investigated in relation to metabolic perturbations [4]. Asian Indians exhibit unique features of obesity; excess body fat, abdominal adiposity, increased SCAT, IAAT, and deposition of fat in ectopic sites (liver, muscle, etc), [5] that may be responsible for high tendency to develop insulin resistance and dysmetabolic state.

It is important to identify cut-offs of SCAT and IAAT for detecting cardiovascular risk, for assessing prognosis and for identification of appropriate therapy. For Asian Indians, the cut-offs for cross-sectional area of total abdominal fat (TAF), SCAT and IAAT as assessed by computerised tomographic scan have been reported recently [6]. It is important to determine the prevalence of high SCAT and IAAT to estimate abdominal adiposity and burden of cardiovascular risk. It is significant to note that there are no data to show high SCAT and IAAT using India specific cut-offs for Indian population. The aim of this study was to assess the prevalence of obesity, abdominal obesity including excess TAF, SCAT and IAAT, the metabolic syndrome and other cardio-metabolic risk factors in urban population in north India.

## Materials and Methods

### Methodology

We performed a cross-sectional, community-based epidemiological study using stratified cluster sampling design in urban New Delhi, India. The study area was divided into approximately four equal sectors using the electoral list. The first house was randomly decided and thereafter every tenth house was taken for the study. If the chosen resident was unwilling to participate in the study, the adjacent house was selected. If desired number of subjects could not be included and the end of the area was reached, investigators returned back to the starting point and the above procedure was repeated until all the remaining subjects were enrolled. The same procedure was applied in all the sectors and sites. A physician, two dieticians, two technicians and two male and female volunteers carried out the study. Of those approached, approximately 80% agreed to participate in the study. Non-participation was uniform in the four sectors and the participation from males and females was approximately equal. All subjects were assessed for demographic and socio-economic profiles, smoking and family history. All the subjects were fully informed about the purpose of the study and a written informed consent was obtained from each of them. Approval for the study was obtained from the institutional ethics committee of All India institute of Medical Sciences (AIIMS), New Delhi.

### Anthropometric measurements

Body weight (to nearest 0.1 kg) and height (to nearest 0.1 cm) were measured while subjects were dressed in light clothing and stood erect with bare foot and eyes directed straight ahead. Body mass index (BMI) was calculated as weight (kg)/ height (m)^2^. Waist circumference (WC) and hip circumference (HC) was measured as previously described [7]. The mean of three readings for each measurement was taken for the calculation of waist-hip ratio (WHR). The skinfolds (biceps, triceps, subscapular and suprailiac) were measured using Lange skinfold calipers by the same physician as previously described [7]. Sum of all skinfolds (Σ4SF) and ratios of subscapular and triceps skinfold (SS/TR ratio) and central (sum of subscapular skinfold and suprailiac skinfold) and peripheral skinfolds (sum of biceps skinfold and triceps skinfold) were calculated.

### Measurement of Percentage Body Fat, Total Abdominal Fat (TAF) and Sub-compartment (IAAT, SCAT)

We estimated percentage body fat (%BF), TAF and areas of abdominal adipose tissue sub-compartments; IAAT and SCAT using the predictive equations developed for Asian Indians which included simple variables such as age, gender, height, weight, BMI, WC, HC, and skinfolds (Table 1) [8]. Cut offs for TAF, IAAT and SCAT developed for Asians were used to determine the abnormal values [8].

**Table 1:** Predictive Equations for Estimation of Body Fat and Abdominal Fat Depots [8].

Variables	Predictive Equation	Cut Offs
%BF^a^	42.42+0.003 × age+7.04 × gender^b^ +0.42 × TR sf^c^ +0.29 × WC^d^ +0.22 × Wt^e^ − 0.42 × Ht^f^	≥25.5% (males) and ≥38% (females)[9]
TAF^g^	−47,657.00+1384.11× gender +1466.54× BMI +416.10× WC	≥245.6 cm^2^ (males) and 203.46 cm^2^ (females)[6]
IAAT^h^	−238.7+16.9×age +934.18×gender +578.09 × BMI – 441.06× HC^i^ +434.2× WC	≥135.3 cm^2^ (males) and 75.73 cm^2^ (females)[6]
SCAT^j^	−49,376.4−17.15× age +1,016.5× gender +783.3× BMI +466× HC	≥110.74 cm^2^ (males) and 134.02 cm^2^ (females)[6]

a Percentage Body fat.

b Male: 1; Female: 2.

c Triceps skinfold.

d Waist circumference.

e Weight.

f Height.

g Total abdominal fat.

h Intra-abdominal adipose tissue.

i Hip circumferences,

j Subcutaneous abdominal adipose tissue.

### Blood Pressure and Biochemical Measurements

Blood pressure, fasting blood glucose (FBG), total cholesterol (TC), serum triglycerides (TG), and high-density lipoprotein cholesterol (HDL-C) were performed as described previously [7].

### Definitions

Overweight and obesity were defined as BMI ≥23–24.9 kg/m^2^ and BMI ≥25 kg/m^2^, respectively [5]. Waist circumference >90 cm for males and >80 cm for females was considered an indicator of abdominal obesity [5]. Cut offs for %BF was taken as 25.5 for males and 38 for females, respectively [9]. Cut offs for TAF [≥245.6 cm^2^ (males) and ≥203.46 cm^2^ (females)], IAAT [≥135.3 cm^2^ (males) and ≥75.73 cm^2^ (females)] and SCAT [≥110.74 cm^2^ (males) and ≥134.02 cm^2^ (females)] developed for Asians were used to determine the adiposity [6]. Further, Σ4SF >50 mm was taken as high [7]. Impaired fasting glucose and T2DM were diagnosed according to the diagnostic criteria of the American Diabetes Association [10]. The modified criteria (three out of five) of National Cholesterol Education Program, Adult Treatment Panel III (NCEP ATP III) were used to define the metabolic syndrome; waist circumference, males >90 cm, females >8 cm, fasting blood glucose >100 mg/dl, serum TG >150 mg/dl, blood pressure >130/85 mmHg and HDL-C; males <40 mg/dl, and females <50 mg/dl [11].

### Statistical methods

Data were recorded on a pre-designed performa. Before entering the data on an Excel spreadsheet, the performa were reviewed for any incomplete information. All the entries were double-checked for any possible keyboard error. For the variables following approximate normal distribution, mean and standard deviation (SD) was computed, while for non-normally distributed variables summary statistics were computed by median and range. Student's t-test was used to compare the mean values in the two independent groups. A p value <0.05 was considered statistically significant. STATA 6∶0 intercooled version (STATA Corp, Houston, Texas, USA) was used for statistical analysis.

## Results

### Demographic Characteristics and Behavioral Determinants

Out of 509 subjects screened, 459 subjects (217 males and 242 females) had complete records. The mean ± SD for age was 42.9±11.7 years. Overall, there was a preponderance of Hindus (92.8%), followed by Sikhs (3.5%), Muslims (3.1%) and Christians (0.6%). The demographic profile and family history of the study population is shown in Table 2.

**Table 2:** Demographic Characteristics and Family History.

Variables n (%)	Total	Males	Females	p value
	n = 459	n = 217	n = 242	
Age (yrs)	42.9±11.7	43.9±12.6	42.1±10.8	0.057
Occupation				
Employed for wages (%)	185 (40.3)	144 (66.4)	41 (16.9)	0.000
Self employed (%)	62 (13.5)	43 (19.8)	19 (7.9)	0.000
Out of work (%)	4 (0.9)	4 (1.8)	0 (0.0)	0.000
Homemaker (%)	178 (38.8)	0 (0.0)	178 (73.6)	0.000
Student (%)	10 (2.8)	8 (3.7)	2 (0.8)	0.000
Retired (%)	20 (4.4)	18 (8.3)	2 (0.8)	0.000
Gross income (`)	23860.4±23690.5	29849.1±25618.2	18490.3 (15904.1 - 21076.5)	0.000
Family History				
High Blood Cholesterol (%)	18 (3.9)	13 (6.0)	5 (2.1)	0.03
Diabetes (%)	109 (23.8)	67 (30.9)	42 (17.4)	0.001
Obesity (%)	73 (15.9)	42 (19.4)	31 (12.8)	0.06
Tobacco Consumption (%)	117 (25.5)	104 (47.9)	13 (5.4)	0.001
Alcohol Consumption (%)	154 (33.6)	149 (68.7)	5 (2.1)	0.001

### Anthropometric and Biochemical measurements (Tables 3, 4, 5)

**Table 3:** Anthropometric and Body Fat Profiles.

Variables	Total	Male	Female	p value
	n = 459	n = 217	n = 242	
BMI (kg/m^2^)	24.9±4.5	24.8±4.1	25.0±4.9	0.7
Waist Circumference (cm)	90.0±12.6	91.6±11.9	88.6±13.0	0.006
Mid Upper Arm Circumference (MUAC) (cm)	28.7±3.5	28.8±3.4	28.7±3.5	0.4
Skinfolds				
Biceps (mm)	13.1±8.5	9.9±6.5	16.0±9.1	1
Triceps (mm)	22.5±9.8	19.1±8.2	25.6±10.2	1
Subscapular (mm)	29.7±12.7	29.4±12.6	29.9±12.7	0.7
Superailiac (mm)	30.0±12.7	28.3±12.4	31.5±12.8	1
Σ4sf (mm)^a^	95.3±38.6	86.7±34.7	103±40.3	1
Central Skinfolds (mm)	59.6±24.0	57.7±23.6	61.4±24.2	1
Peripheral Skinfolds (mm)	35.6±17.7	29.0±14.1	41.6±18.4	1
Central-Peripheral skinfold ratio	1.8±0.7	2.1±0.7	1.6±0.5	0.000
SS/TR ratio b	1.4±0.6	1.6±0.6	1.2±0.5	0.000
% Body Fat	33.43±11.03	29.4±8.9	40.8±10.0	1
Total Abdominal Fat (cm^2^)	284.1±114.9	281.31±105.9	286.6±122.5	0.69
Intra Abdominal Adipose Tissue (cm^2^)	132.7±53.6	138.01±49.2	127.9±56.9	0.02
Subcutaneous Abdominal Adipose Tissue (cm^2^)	154.3±72.4	143.4±64.3	164.1±77.8	0.99

All values in mean and SD,

a Σ4sf; Sum of 4 skin folds (Biceps, Triceps, Subscapular, Superailiac),

b SS/TR; Sub scapular-Triceps skinfold ratio.

**Table 4:** Prevalence of Obesity and Regional Adiposity.

Variables	Total	Male	Female	p value
	n = 459 (%)	n = 217 (%)	n = 242 (%)	
Overweight	76 (16.5)	40 (18.4)	36 (14.9)	0.5
Obesity (according to BMI)^a^	230 (50.1)	109 (50.2)	121 (50.0)	0.5
Obesity (according to % Body Fat)^a^	388 (84.5)	146 (67.2)	242 (100)	0.000
Abdominal Obesity (according to WC)^a^	316 (68.9)	135 (62.2)	181 (74.8)	0.004
TAF (cm^2^)^b^	325 (70.8)	146 (67.3)	179 (73.97)	0.116
IAAT (cm^2^)^c^	318 (69.3)	123 (56.7)	195 (80.6)	0.000
SCAT (cm^2^)^d^	311 (67.8)	150 (69.1)	161 (66.5)	0.5
Σ4sf (mm)^e^	392 (85.4)	178 (82.0)	214 (88.4)	0.05

a Please refer to text for definition and cut offs.

b TAF: Total Abdominal Fat,

c IAAT; Intra Abdominal Adipose tissue,

d SCAT; Subcutaneous Adipose Tissue,

e Σ4sf; sum of 4 skinfolds (biceps, triceps, subscapular and superailiac).

**Table 5:** Prevalence of Cardio-metabolic Risk Factors.

Variables	Total	Male	Female	p value
	n = 459 (%)	n = 217 (%)	n = 242 (%)	
Impaired Fasting Glucose	110 (24.0)	57 (26.3)	53 (21.9)	0.5
Diabetes	39 (8.5)	19 (8.8)	20 (8.3)	0.85
The Metabolic Syndrome	208 (45.3)	71 (32.7)	137 (56.6)	0.000
Hypertension	134 (29.2)	73 (33.6)	61 (25.2)	0.047
Hypercholesterolemia	122 (26.6)	68 (31.3)	54 (22.3)	0.29
Hypertriglyceridemia	196 (42.7)	107 (49.3)	89 (36.8)	0.007
LDL-c ≥100 mg/dL	237 (51.6)	116 (53.5)	121 (50.0)	0.46
HDL-c<40 mg/dL (males) <50 mg/dL (females)	170 (37)	9 (4.2)	161 (66.5)	0.000

LDL-c; Low density Lipoprotein cholesterol, HDL-c; High density Lipoprotein cholesterol.

The prevalence of obesity was 50.1%, and abdominal obesity by WC and TAF was 68.9% and 70.8%, respectively. Excess IAAT and SCAT were seen in 69.3% and 67.8% subjects, respectively. There was a high prevalence of hypertension, hypercholesterolemia, hypertriglyceridemia, low HDL, the metabolic syndrome, impaired fasting glucose (IFG) and diabetes.

## Discussion

This is the first paper on Asian Indians showing high prevalence of abdominal adiposity and excess adiposity in various abdominal sub compartments using predictive equations for body fat and abdominal fat developed for Asian Indians. The prevalence of obesity (by BMI) was 50.1% in the current paper which is comparable to the prevalence of 45.9% and 55.5% reported in urban populations of Chennai (South India) [12] and Jaipur (north India) [13], respectively. High prevalence of obesity based on percentage body fat (84.5%) was notable. The conspicuous feature in women, therefore, was under-representation of obesity when defined by BMI alone. These observations are of considerable practical relevance, questioning BMI as a valid epidemiological tool in Asian Indian population, particularly in females.

The prevalence of abdominal obesity as assessed by WC (68.9%) was similar to that assessed by TAF (70.8%). The study showed high prevalence of intra abdominal (69.3%) and subcutaneous adiposity (67.8%). Thus, about 70% of the population having abdominal obesity as assessed by multiple parameters in the current study is of considerable concern because of associated metabolic and cardiovascular consequences. Of significance, the prevalence of abdominal obesity in this study is substantially higher to that reported in urban population of Chennai (46.6%) [12]. Further, the prevalence of abdominal obesity in the current study was higher in women (74.8%) than men (62.2%; p<0.01) which is comparable to studies done in north (57.3% in men, 68% in women) [14] and South India (35.1% in men, 56.2% in women) [12].

Studies focusing on total adiposity, regional fat depots use methods like dual-energy X-ray absorptiometry (DEXA) scan, computerized tomography or magnetic resonance imaging (MRI) scans using special software to quantify adiposity. These methods are expensive, available in selected hospitals in metropolitan cities, and the software is available in only a few centers in India. In this study we used, equations to predict % BF, TAF, SCAT, and IAAT using simple anthropometric variables such as age, gender, BMI, WC, HC, and skin folds, for the first time in Asian Indians. The mean value of IAAT (132.7±53.6 cm^2^) and SCAT (154.3±72.4 cm^2^) in the present study were higher than the values reported in our previous study done on healthy adults (IAAT; 80.0±67.8 cm^2^ , SCAT; 100.6±68.2 cm^2^) [15]. Similarly in the present study the mean IAAT value was higher than that reported in urban, non-diabetic Asian Indians residing in South India however the mean SCAT value was much lower (IAAT;119.3±33.3 cm^2^, SCAT; 208.7±118.6 cm^2^) [16]. Further in the present study the mean value for IAAT were higher in males (p = 0.02) while the mean values for SCAT were higher in females (p = 0.99) which was similar to that seen in urban Asian Indians residing in South India (IAAT p = 0.267; SCAT p <0.001 for males and females, respectively) [17].

Several studies have shown that both IAAT and SCAT are associated with adverse cardiometabolic risk factors [18], [19]. Increased visceral fat is related to dyslipidemia and increased frequency of insulin resistance and may account for the increased prevalence of diabetes mellitus and coronary artery disease in Asian Indians [20] while increased truncal skinfold thickness (indicative of truncal subcutaneous adipose tissue) independently predicts cardiovascular risk [21]. Importantly, the ratio of subscapular to triceps skinfold and abdominal obesity was shown to be independently associated with surrogate markers of insulin resistance and type 2 diabetes in Hispanic populations [22]. Adult South Asians and post-pubertal children have thicker truncal skinfolds than similar populations of white Caucasians [23], [24]. Among abdominal adipose tissue depots, SCAT as compared to IAAT, is more significantly associated with the metabolic syndrome in Asian Indians living in India [15]. Regardless of these observations and continuing debate regarding metabolic importance of IAAT *vs.* SCAT, it appears that both contribute significantly to metabolic and cardiovascular risk. However, it is important to note that due to higher mass of SCAT than IAAT, it may affect metabolic factors more significantly. In this context, it is matter of concern that substantial percentage of women in the current study had both high SCAT and IAAT, while men fared slightly better in latter.

It is understandable that with such high prevalence of abdominal adiposity, co-morbid risk factors, dysglycemia and dyslipidemia would be high. Of specific concern is presence of high prevalence of hypertriglyceridemia (42.7%) in the current study, which is higher than that reported in urban population of Chennai (34.1%) [25], but was comparable to another study in Chennai (41.1%) [26]. Overall high prevalence of the metabolic syndrome (45.3%) in the current study is similar to that seen in urban population of Chandigarh in north India (45.3%) [27] but was higher than that reported in urban Mumbai (35.2%) [28] and Chennai (34.1%) [25]. In the present study the prevalence of the metabolic syndrome was significantly higher in females than males, which has been repeatedly reported from India [26], [29].

There are some limitations to our study. Being a cross-sectional study, no cause/effect inferences can be drawn. Secondly, for estimation of %BF, TAF, IAAT, and SCAT we used the predictive equations and not imaging techniques.

In summary, high prevalence of obesity, and abdominal obesity (as shown by various measures) and high prevalence of coexistent cardiovascular risk factors in the urban population of New Delhi is of concern, and need application of primary prevention strategies.
